# Supplementary figures and images for: Productive HIV-1 infection of tissue macrophages by fusion with infected CD4+ T cells
Source: J Cell Biol. 2023 Mar 29;222(5):e202205103. doi: 10.1083/jcb.202205103 (PMC10067447; doi:10.1083/jcb.202205103)

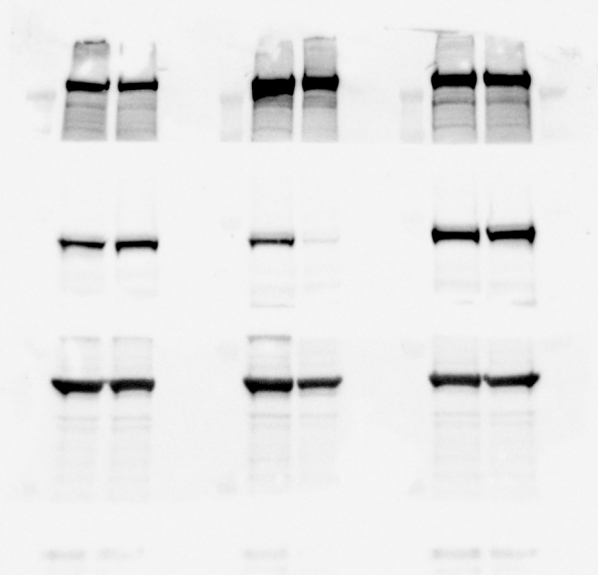

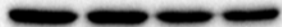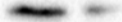

D360 nbA

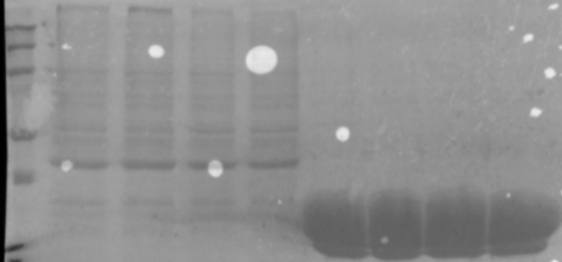

70

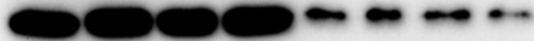

Supplement: SourceData FS5 — is the source file for Fig. S5. [file JCB_202205103_SourceDataFS5.pdf]

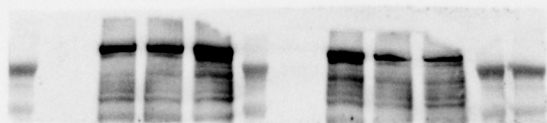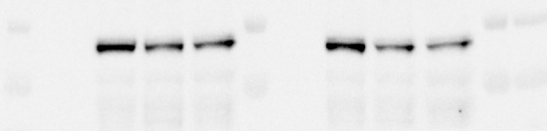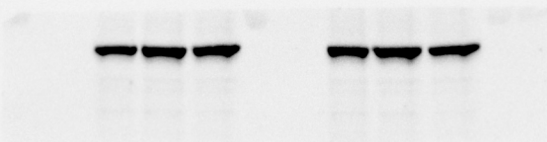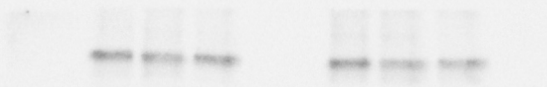

Supplement: SourceData FS7 — is the source file for Fig. S7. [file JCB_202205103_SourceDataFS7.pdf]
